# Plasma glycocholic acid and linoleic acid identified as potential mediators of mitochondrial bioenergetics in Alzheimer’s dementia

**DOI:** 10.3389/fnagi.2022.954090

**Published:** 2022-09-23

**Authors:** K. Allison Amick, Gargi Mahapatra, Zhengrong Gao, Amber Dewitt, Suzanne Craft, Mohit Jain, Anthony J. A. Molina

**Affiliations:** ^1^Section on Gerontology and Geriatrics, Department of Internal Medicine, Sticht Center for Healthy Aging and Alzheimer’s Prevention, Wake Forest School of Medicine, Winston-Salem, NC, United States; ^2^Department of Neuroscience, Wake Forest School of Medicine, Winston-Salem, NC, United States; ^3^Department of Pharmacology, School of Medicine, University of California, San Diego, La Jolla, CA, United States; ^4^Division of Geriatrics, Gerontology, and Palliative Care, Department of Medicine, School of Medicine, University of California, San Diego, La Jolla, CA, United States

**Keywords:** Alzheimer’s disease, mitochondria, metabolomics, plasma, bioenergetics, neurons, glycocholic acid, linoleic acid

## Abstract

Mitochondrial bioenergetic alterations occur in the brain and peripheral cells of patients with Alzheimer’s disease (AD). This study focuses on plasma circulating factors, namely lipids, as mediators of systemic bioenergetic differences in participants with normal cognition (NC), mild cognitive impairment (MCI), and dementia due to probable AD (DEM). We examined bioenergetic differences across cognitive groups by measuring the mitochondrial respiration of peripheral blood mononuclear cells (PBMCs) from 37 participants (12 NC, 12 MCI, 13 DEM). PBMC bioenergetics were lower in the DEM group compared to the NC group. To determine whether circulating factors can mediate bioenergetic differences according to cognitive status, we exposed naïve neuronal Neuro-2a (N2a) cells to plasma from each participant *in vitro*. N2a bioenergetics were lower following plasma exposure from DEM compared to NC group participants. Notably, PBMC Max and N2a Max positively correlated, suggesting that circulating factors modulate the bioenergetics of naïve N2a cells according to the bioenergetic capacity of donor primary PBMCs. To identify lipid metabolites that may contribute to bioenergetic differences between cognitive groups, we performed liquid chromatography-mass spectrometry to assess the abundance of individual lipid species and correlated PBMC and N2a bioenergetics. Glycocholic acid (GCA) positively correlated with PBMC and N2a bioenergetics, while linoleic acid (LA) was negatively correlated. These data suggest that GCA and LA may contribute to the stimulatory and inhibitory bioenergetics effects related to cognitive status. *Post hoc* analyses revealed that GCA abundance was lower by 52.9% in the DEM group compared to the NC group and that LA abundance was higher by 55.7% in the DEM group compared to the NC group. To validate these findings, we examined the abundance of GCA and LA in the larger, more diverse, parent cohort (*n* = 378) and found similar results; GCA abundance was lower by 29.7% in the DEM group compared to the NC group and LA abundance was higher by 17.8% in the DEM group compared to the NC group. These data demonstrate that circulating factors have a direct effect on mitochondrial bioenergetics and that individual circulating factors identified to be associated with mitochondrial function are differentially expressed in patients with dementia.

## Introduction

In 2004, Swerdlow and Khan (2004) posed the mitochondrial cascade hypothesis to explain the development of sporadic late-onset Alzheimer’s Dementia (AD) based on mounting evidence that mitochondrial dysfunction preceded deposition of amyloid β plaques and neurofibrillary tangles. Mitochondrial dysfunction is now recognized to occur early in AD and accompanies disease progression (Yao et al., 2009; Swerdlow, 2011; Caldwell et al., 2015).

Mitochondrial dysfunction also occurs in the peripheral cells and tissues of patients with AD. Compared to healthy controls, platelets from patients with AD have decreased cytochrome oxidase activity (Parker et al., 1990; Parker et al., 1994). Fibroblasts from AD patients exhibit altered mitochondrial function and have increased glycolytic capacity, lower glutamine metabolism, and abnormal mitochondrial metabolic potential (Sims et al., 1987; Sonntag et al., 2017). Peripheral circulating blood cells, such as mixed peripheral blood mononuclear cells (PBMCs), monocytes, and platelets also experience decreases in mitochondrial components and respiration in other disease states (Parker et al., 1989; Sangiorgi et al., 1994; Tyrrell et al., 2015; Michalak et al., 2017; Chacko et al., 2019), can report on bioenergetics in the brain independent of AD (Tyrrell et al., 2017), and are related to white matter, gray matter, and total intracranial volumes independent of AD (Mahapatra et al., 2018). We posit that these circulating cells are likely responding to continuous exposure to circulating factors in the blood, some of which may be “mito-active,” having direct effects on mitochondrial function.

Among the various classes of circulating factors, lipids are of particular interest. Several studies comparing normo-cognitive and cognitively impaired individuals have found differences in circulating non-cellular lipids. For instance, a study of 123 individuals divided into AD, MCI, and elderly control groups demonstrated that the abundance of just 10 lipid metabolites could identify patients from healthy controls (Proitsi et al., 2015). In another study, several species of sphingomyelin were decreased and 2 ceramide species were increased in early AD plasma compared to controls (Han et al., 2011). Moreover, Wong et al. (2019) and Liu et al. (2021) reported that Aβ is contributory, if not causal, in the differences observed in peripheral lipidomes between cognitive groups and Wong et al. (2019) describe differences in plasma lipidomes in healthy older adults based on apolipoprotein E status, a protein that binds and carries lipids and increases or decreases the risk of developing AD depending on the isoform. Despite mounting evidence for the importance of circulating factors, and lipids in particular in the pathophysiology of AD, the identities of plasma factors that affect mitochondrial bioenergetics in the context of AD are poorly understood.

In order to address the effect of circulating lipids on bioenergetics in AD, we measured the bioenergetics of circulating PBMCs using both intact and permeabilized cells of 37 participants divided into cognitively normal (NC), mild cognitive impairments (MCI), or dementia due to probable AD (DEM) to examine systemic differences in bioenergetics between cognitive groups. We then incubated naïve N2a cells with fibrinogen-depleted plasma from the same blood donors and measured respiration to evaluate if non-cellular circulating factors can mediate bioenergetic differences in naïve cells. Corresponding plasma samples from each participant were then analyzed by liquid chromatography-mass spectrometry (LC-MS) to determine the abundance of plasma lipids. The identification of candidate “mitoactive” lipids was based on correlations analysis comparing the abundance of each lipid with the PBMC Max and N2a Max. We identified 2 candidate lipid molecules that met both of these predetermined selection criteria. *Post-hoc* tests for candidate lipids evaluated differences in abundance across cognitive groups and correlations with cognitive performance. To validate the 2 lipid candidates identified from our 37-participant cohort, we examined the abundance of these lipids in plasma samples from a larger, more diverse, 378-participant cohort that included NC, MCI, and DEM participants.

## Materials and methods

### Participants

The Wake Forest School of Medicine Institutional Review Board approved all procedures and written informed consent was obtained from all participants and/or their legal representative. Plasma and bioenergetic data were collected from a subcohort of 37 participants enrolled in the Wake Forest Alzheimer’s Disease Research Center’s (WF ADRC) Alzheimer’s Disease Clinical Core (ADCC) cohort. Sample size was determined by availability of samples and feasibility of experiments. However, the study was adequately powered to detect differences between cognitive groups. Power calculations for each cognitive measure reported that the current sample size is powered > 0.99 for the current effect sizes, indicating that these are three distinct groups when using these samples. Participants were excluded if they were younger than 55 years of age, had other reasons for impaired cognition such as significant neurologic disease other than Alzheimer’s disease or use of sedating medication as judged by study clinicians, had a clinically significant medical illness, had a current or recent substance abuse disorder, had a poorly controlled psychiatric disorder, or were currently using insulin. Participants were evaluated by clinical and cognitive assessment and received a brain MRI in order to determine clinical diagnosis. Weekly multidisciplinary consensus conferences assessed the clinical, cognitive, and MRI data of each participant the determine assignment to one of three groups: normo-cognitive, MCI, or dementia using the current National Institute of Aging-Alzheimer’s Association guidelines for the diagnosis of MCI and dementia (Albert et al., 2011; McKhann et al., 2011).

All participants underwent dementia evaluation using version 3 of the Uniform Data Set (a uniform set of procedures for characterizing individuals with mild AD and MCI as compared to aging without dementia used by the Alzheimer’s Disease Centers) upon enrolling in the study (Morris et al., 2006; Besser et al., 2018). Briefly, this included recording health history, receiving a physical examination by a study clinician experienced in dementia care, assessment of function in work or usual activities including signs of functional decline using the Clinical Dementia Rating scale (Morris, 1993), screening for neuropsychiatric symptoms and depression, and completing a comprehensive neuropsychological testing battery (Weintraub et al., 2018). The Free and Cued Selective Reminding Test (FCSRT) (Grober et al., 2009), Digit Symbol Substitution Task (DSST) (Kaufman, 1983), and the Mini-Mental State Exam (MMSE) (Folstein et al., 1975) were also administered as supplemental cognitive assessments.

A composite measure of cognitive function, the Modified Preclinical Alzheimer’s Cognitive Composite (mPACC5), was calculated for all participants. The standard Preclinical Alzheimer’s Cognitive Composite (PACC5) combines scores from five separate cognitive tests: MMSE, FCSRT free and total (FCSRT96; 0-96), Logical Memory Delayed Story Recall (Wechsler, 1987), DSST, and category fluency (Monsch et al., 1992). We modified the PACC5 to create the mPACC5 by substituting comparable tasks based on those available in the WF ADRC cognitive testing battery. The Craft Story verbatim recall (Craft et al., 1996) was used in place of Logical Memory Delayed Story Recall and FCSRT total recall (FCSRT48; 0–48). We measured the mean and standard deviation in cognitively normal participants for each of these five tests to calculate a z-score across all participants. The average z-score was used to produce a Modified Preclinical Alzheimer’s Cognitive Composite (mPACC5) score.

Hemoglobin H1c (HbA1c) was measured through standard whole blood collection. Current criteria define HbA1c values that are ≥ 6.5% as diabetes and 5.7–6.4% and prediabetes, Impaired glucose tolerance was defined as meeting the criteria for either diabetes or prediabetes.

### Peripheral blood mononuclear cell isolation

Participants fasted overnight prior to collection of 16 mL of venous blood in two acid citrate dextrose tubes (8 ml per tube) (02-684, BD Vacutainer™; Becton Dickinson, Franklin Lakes, NJ) to minimize immediate effects of diet on bioenergetics. Samples were transported at room temperature at all times to avoid heating and cooling and were processed immediately. All assays were performed at the same temperature and almost exclusively by a single laboratory personnel within an hour of blood draw. Previously published methods were used to isolate PBMCs (Tyrrell et al., 2015; Mahapatra et al., 2018). Briefly, whole blood was centrifuged at 500 × g for 15 min in the acid citrate dextrose tubes with no brake at room temperature. The buffy coat layer containing PBMCs was extracted and diluted in RPMI 1640 (11835030, Gibco, Grand Island, NY) containing 100 μM prostaglandin E_1_ (PGE1) (13010, Cayman Chemical, Ann Arbor, MI). The PBMC/RMPI dilution was divided into three parts and each was layered onto 3 mL of 1.077 g/mL density Histopaque^®^-1077 (10771, Sigma-Aldrich, St. Louis, MO) in 15 mL centrifuge tubes. The layered solution was centrifuged at 700 × g for 30 min with no brake. The hazy PBMC layer was extracted and diluted up to 25 mL using RPMI 1640 containing 200 nM PGE1. The PBMC/RMPI dilution was then centrifuged at 500 × g for 5 min with the brake on. The resulting pellet was resuspended in 25 mL RPMI 1640 containing 200 nM PGE1 and centrifuged at 500 × g for 5 min with the brake on. PBMCs pellet was resuspended in extracellular flux (XF) assay buffer (102353, Agilent, Santa Clara, CA) containing 1 mM Na^+^ pyruvate (11360070, Gibco), 1 mM glutaMAX (35050061, Gibco), 11 mM D-(+)-glucose (G8769, Sigma-Aldrich), and 1 μM PGE1. PBMCs were counted using a Coulter AC Tdiff2 (Beckman Coulter, USA) to ensure consistency in cell counting. The Coulter AC Tdiff2 is calibrated once yearly per the vendor’s recommendations and is accuracy is tested weekly with control reagents.

### Peripheral blood mononuclear cell respirometry

Respirometric analysis of intact PBMCs isolated on the same day was performed using an Agilent/Seahorse XF^e^24 extracellular flux analyzer (XF^e^24) (Agilent, Inc., Santa Clara, CA). PBMCs were plated in quadruplicate at 250,000 cells/well in an XFe24 assay plate and incubated for 1 h at 37°C, atmospheric CO_2_. The XF media contained 1 mM glutaMAX, 1 mM sodium pyruvate, and 11 mM D-glucose. The mitochondrial stress test was performed with final in-well concentrations of 0.75 μM oligomycin (Sigma-Aldrich) or 20 μL of plain media in port A, 1.0 μM FCCP (carbonyl cyanide-4- (trifluoromethoxy) phenylhydrazone; Sigma-Aldrich) in port B, and 1.0 μM Antimycin A (Sigma-Aldrich) and 1.0 μM Rotenone (Sigma-Aldrich) in port C. The third measurement was used to calculate PBMC Basal Respiration, respiration levels at the start if the assay before cells have been exposed to inhibitors (oligomycin, antimycin A, or rotenone) or the uncoupler (FCCP). The third measurement following oligomycin was used to calculate PBMC Leak respiration. Oligomycin inhibits ATP synthase activity. Any respiration is due to “leak” in the ETS as protons should not be moving between the matrix and the intermembrane space when ATP synthase is inhibited. The greatest of the three values following the FCCP addition was used to calculate PBMC Max respiration as FCCP uncouples the ETS from ATP Synthase activity by allowing protons to move across the inner membrane from the matrix to the intermembrane space from the matrix so that ATP synthase is free to pump protons into the matrix at an uninhibited rate. Non-mitochondrial respiration was subtracted from Basal, Leak, and Max respiration values. PBMC Spare Respiratory Capacity (SRC) was calculated by subtracting Basal from Max respiration and represents the additional respiratory capacity available between Basal and Max respiration. PBMC ATP-linked respiration was calculated by subtracting Leak from Basal respiration. The difference between Basal and Leak represents the respiration that is specifically linked to ATP production. Any wells that diverged by ± 20 from 150 mmHg, while not background corrected, experienced oxygen saturation that did not return to pre-measurement saturations, or did not respond within 75% of the average change of the other wells in the same group were excluded. The Seahorse bioanalyzer is also calibrated before each assay.

**FIGURE 1:**
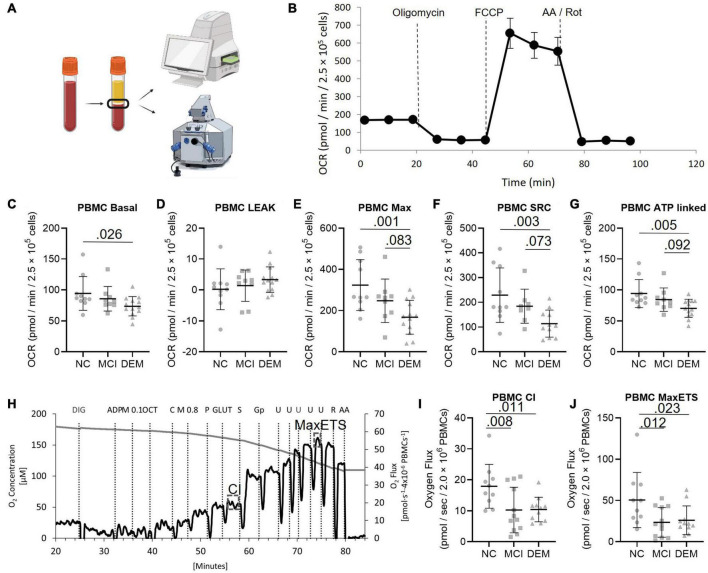
PBMC bioenergetics from NC, MCI, and DEM participants. **(A)** Schematic of bioenergetic workflow. **(B)** Representative Seahorse XF24^e^ bioenergetics trace. **(C–G)** Basal, Leak, Max, SRC, and ATP-linked by cognitive status. **(H)** Representative O2k trace. CI uses saturating concentrations of octanoylcarnitine, malate, pyruvate, and glutamate as substrates. Max ETS is achieved using saturating concentrations of octanoylcarnitine, malate, pyruvate, glutamate, succinate (for complex II), glycerol-3-phosphate (for glycerol-3-phosphate dehydrogenase), and then titrated with FCCP to uncouple respiration to achieve maximal respiration. **(I,J)** Complex I and MaxETS respiration by cognitive status. Exact *P*-values reported.

High-resolution permeabilized PBMC respirometry on cells isolated the same day was performed using an Oroboros Oxygraph-2K (Oroboros, Innsbruck, Austria) with 2 × 10^6^ cells per chamber. The previously published substrate-uncoupler-inhibitor titration (SUIT) reference protocol 2 was modified and used to measure O_2_ Flux in pmol⋅s^–1^⋅2 million^–1^ cells. Respirometry chambers were filled with 2 mL of mitochondrial respiration medium (MiR05) made with 0.5 mM EGTA, 3 mM MgCl_2_, 60 mM lactobionic acid, 20 mM taurine, 10 mM KH_2_PO_4_, 20 mM HEPES, 110 mM D-Sucrose, and 1 g/L fatty acid free BSA, pH 7.1. Chambers were equilibrated at room oxygen concentration at 37°C for at least 30 min for daily oxygen calibration. PBMCs were then added to the chamber, the stopper was closed, and the O_2_ Flux was allowed to equilibrate. PBMCs were permeabilized using 0.04 mg/mL digitonin. Cell permeabilization was followed by sequential addition of 1 mM ADP and 0.6 mM Mg, 0.1 mM malate, 0.5 mM octanoylcarnitine, 0.01 mM cytochrome c, 2 mM malate, 5 mM pyruvate, 10 mM glutamate, 10 mM succinate, 10 mM glycerophosphate, 0.5 μM FCCP titrations until maximal ETS capacity was achieved, 1 μM rotenone, and 2.5 μM antimycin A. All concentrations are reported as final concentrations inside the respirometric chamber. PBMC Complex I (CI) respiration was measured following the glutamate injection and PBMC MaxETS was measured following the greatest bioenergetic value after FCCP titration. The hashed boxes on the high-resolution respirometry trace mark the bioenergetic parameters measured during this assay (Figure 1H). Before analysis, residual oxygen consumption (ROX) was subtracted from each bioenergetic parameter. Any assays that ended with a negative value following the antimycin A injection were excluded from the dataset. Mitochondrial integrity following permeabilization was also tested by evaluating the cytochrome c response. Samples that had an increase in respiration by more than 10% following cytochrome c addition were not used. Additionally, a cell viability with protocol described in our recently accepted publication added further validation to confirm mitochondrial integrity (Mahapatra et al., 2022). For additional information on the terms and abbreviations adopted to describe the high-resolution respirometry parameters in this manuscript refer to Gnaiger (2014).

### Cell culture

Neuro-2a (N2a) cells (CCL-131, ATCC) were cultured in DMEM (11960044, Thermo Fisher, Waltham, MA) supplemented with 10% fetal bovine serum, 1% penicillin-streptomycin at 10,000 U/mL (15140122, Gibco), and 2 mM glutaMAX. All experiments were conducted between passage 4 and passage 9. There were no differences in morphology or bioenergetics noted between passages. Cells were cultured at 37°C and 5% CO_2_.

### Fibrinogen-depleted plasma preparation

**FIGURE 2:**
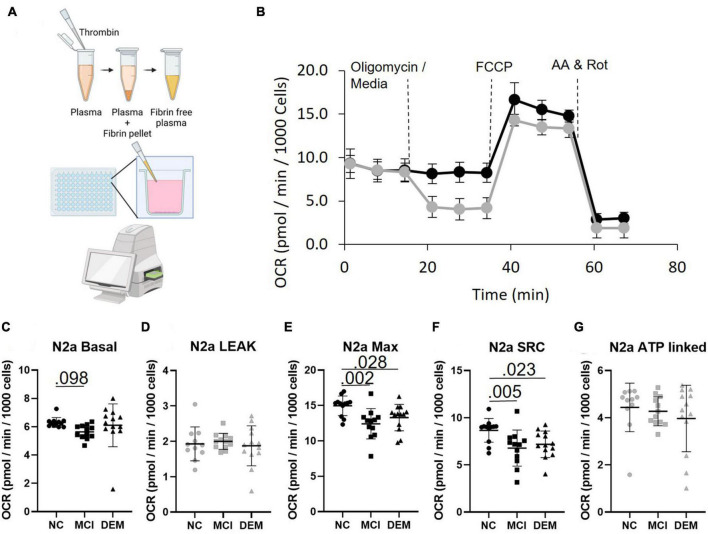
Bioenergetics of N2a cells treated with fibrinogen-depleted plasma from NC, MCI, and DEM participants. **(A)** Schematic of bioenergetic workflow. **(B)** Representative Seahorse XF96^e^ trace. The gray line includes an oligomycin injection and the black line excludes the oligomycin injection. Measures of Leak and ATP-linked (plots have gray markers) are calculated from the assay with oligomycin. Measures of Basal, Max, and SRC (plots have back markers) are calculated from the assay without oligomycin, as oligomycin had an inhibitory effect on Max Respiration. **(C–G)** Basal, Leak, Max, SRC, and ATP-linked respiration by cognitive status. Exact *P*- values reported.

The day before using the plasma in an assay, plasma samples from each participant were thawed in 250 μL aliquots in a 1.5 mL centrifuge tube on ice for 3 h. Each sample was then treated with 2 μL of native human thrombin protein (ab62452, Abcam) to cleave fibrinogen into fibrin to prevent coagulation in the cell culture (Figure 2A). The plasma/thrombin mixture was mixed by manually flicking the tube until a visible fibrin strand formed in the bottom of the tube. This mixture was left to sit overnight at 4°C. The following day, the fibrinogen-depleted plasma was transferred to a new tube for use in assays.

### Plasma incubation and Neuro-2a respirometry

N2a cells were plated in an XF^e^96 assay plate at 10,000 cells/well in 160 μL culture media and allowed to rest for 1 h before incubating at 37°C, 5% CO_2_ for 24 ± 1 h. Following the 24-h incubation, 140 μL of culture media was removed from each well, with care not to scrape cells adhered to the bottom of the well. Cells were washed with 140 μL culture media containing 1% Penicillin Streptomycin, and 200 μM glutaMAX to remove FBS. 160 μL of culture media containing 10% fibrinogen-depleted plasma from a single participant instead of FBS was then added per well in quadruplicate. This was executed with plasma from each participant. Culture media containing 10% participant fibrinogen-depleted plasma was then exchanged for XF media containing 2 mM glutaMAX, 1 mM sodium pyruvate, and 25 mM D-glucose and incubated for 1 h at 37°C, atmospheric CO_2_. The Seahorse assay was then run with final in-well concentrations of 0.5 μM oligomycin or 20 μL of plain media in port A, 0.75 μM FCCP in port B, and 1.0 μM Antimycin A and 1.0 μM Rotenone in port C (Figures 2A,B). Two assays were run per individual, with and without oligomycin as oligomycin inhibits Max respiration, but is necessary for Leak and ATP-linked respiration.

### Lipid metabolite extraction

Lipids were isolated from 20 μL of plasma from each participant. Briefly, the 20 μL of plasma was added to 80 μL of 50:50 ethanol: water. Lipids were extracted using a solid-phase system, the Strata-X polymeric 10 mg/mL 96-well SPE plate. Samples were then put through 3 freeze-thaw cycles, alternating between 37°C and –80°C liquid baths in 30-s intervals, followed by vortexing the sample, and 2-min of sonication. Samples were then placed in a –20°C freezer for 30 min, vortexed for 60 s, and centrifuged at 14,000 g for 10 min at 4°C. The supernatant was collected and moved to LC-MS vials.

### Liquid chromatography-mass spectrometry metabolomics analysis

LC-MS metabolomics was performed using a Vanquish UPLC coupled to a high-resolution QExactive orbitrap spectrometer (Thermo Fisher). For chromatographic separation of small polar, bioactive lipids, a Phenomenex Kinetex C18 (1.7 μm particle size, 100 × 2.1 mm) column was used and maintained at 50^°^C using mobile phases A (70% water, 30% acetonitrile, and 0.1% acetic acid) and B (50% acetonitrile, 50% isopropanol, 0.02% acetic acid) ran with the following gradient: 1% B from –1.00 to 0.25 min, 1–55% B from 0.25 to 5.00 min, 55–99% B from 5.00 to 5.50 min, and 99% B from 5.50 to 7.00 min.

A Thermo Q-Exactive orbitrap mass spectrometer was operated in negative mode only for bioactive lipid analysis. High-resolution parent mass spectra were collected over a mass range of 50–1,500 *m/z* negative ionization mode to capture the widest possible range of metabolite chemistries. Spectral data were rigorously examined using quality control and assurance workflows to assess for fluctuations in extraction efficiency, sample degradation, and/or LC-MS system performance drift. All spectral peaks were extracted and quantified using a combination of MzMine and custom in-house software. Metabolites were identified definitively using MS/MS analysis, with the matching of LC measures and MS/MS fingerprint to a custom, in-house database of over 2,000 metabolite standards.

### Statistical analysis

Differences between cognitive groups for PBMC bioenergetics, N2a bioenergetics, and lipid abundance were assessed using a univariate general linear model; the validation group also used age, ApoE status, and sex as covariates (SPSS version 22; Armonk, NY). Normality of respirometric and lipid abundance values was tested using D’Agostino and Pearson test (GraphPad Software, Inc., San Diego, CA). Relationships between lipid abundance and PBMC bioenergetics, N2a bioenergetics, and cognitive scores were established using Spearman correlations (GraphPad Software, Inc., San Diego, CA). Exact *P*-values and *r*-values are reported.

## Results

### Characteristics of the study cohort

Discovery of mitoactive lipids in human plasma focused on 37 representative participants from the larger WF ADRC ADCC cohort. The participants were divided into 3 cognitive groups: NC, MCI, and DEM. Cognitive groups are age-matched (*P* = 0.886) and BMI matched (*P* = 0.669) with differences in 3 cognitive scores: MOCA (*P* < 0.0001), MMSE (*P* < 0.0001), and mPACC5 (*P* < 0.0001) (Table 1). Pairwise comparisons between the cognitive groups for age, BMI, and HbA1c were not different between cognitive groups. Pairwise comparisons between the cognitive groups revealed minor or no differences between cognitive scores for NC vs. MCI, but major differences between NC vs. DEM and MCI vs. DEM (Supplementary Table 1).

**TABLE 1:** Demographics and cognitive scores for discovery cohort.

	Average (SD)			One-way ANOVA
	NC (*n* = 12)	MCI (*n* = 12)	DEM (*n* = 13)	*P*-value
Age, *y*	73.9 (5.3)	73.5 (3.4)	74.5 (6.6)	0.886
BMI	25.4 (3.8)	26.6 (3.4)	26.7 (4.6)	0.669
Female, %	66	58	58	
MMSE, score	28.7 (1.3)	28.0 (1.8)	21.8 (4.9)	<0.0001
MOCA, score	26.2 (2.0)	23.3 (2.6)	18.0 (3.8)	<0.0001
mPACC5, score	–0.18 (0.43)	–1.11 (1.87)	–4.99 (2.48)	<0.0001
HbA1c	5.4 (0.40)	5.5 (0.21)	5.5 (0.46)	0.807

HbA1c, Hemoglobin A1c. For HbA1c parameter: NC, n = 4; MCI, n = 9; DEM, n = 11.

The validation portion of this study focused on 365 participants from the same WF ADRC ADCC cohort and used the same cognitive groupings as the discovery cohort. Cognitive groups exhibited differences in average age (*P* < 0.0001) were BMI matched (*P* = 0.292) and had differences in 3 cognitive scores: MOCA (*P* < 0.0001), MMSE (*P* < 0.0001), and mPACC5 (*P* < 0.0001) (Table 2). Pairwise comparisons between the cognitive groups for BMI, and HbA1c were not different between cognitive groups. Pairwise comparisons between the cognitive groups revealed major differences between age and cognitive scores for NC vs. MCI, NC vs. DEM, and MCI vs. DEM (Supplementary Table 2).

**TABLE 2:** Demographics and cognitive scores for validation cohort.

	Average (SD)			One-way ANOVA
	NC (*n* = 188)	MCI (*n* = 138)	DEM (*n* = 39)	*P*-value
Age, *y*	68.7 (8.0)	71.2 (7.5)	74.5 (8.0)	<0.0001
BMI	27.8 (5.7)	28.2 (5.5)	26.6 (4.3)	0.292
Female,%	77.0	57.2	46.2	
MMSE, score	28.9 (2.3)	27.2 (2.0)	22.9 (4.5)	<0.0001
MOCA, score	26.4 (2.5)	21.7 (3.3)	17.1 (4.9)	<0.0001
mPACC5, score	0.0088 (0.50)	–1.24 (1.49)	–4.51 (3.09)	<0.0001
HbA1c	5.6 (0.46)	5.8 (0.73)	5.6 (0.46)	0.124

HbA1c, Hemoglobin A1c. For HbA1c parameter: NC, n = 103, MCI = 91, and DEM = 31.

### Peripheral blood mononuclear cell bioenergetics are different between cognitive groups

We compared the bioenergetic profiles of PBMCs isolated from whole blood of individuals in the NC, MCI, and DEM groups (Figure 1A) using a mitochondrial respirometry with intact cells (Figure 1B), and a high-resolution respirometry substrate-uncoupler-inhibitor titration (SUIT) protocol with permeabilized cells (Figure 1G).

In intact cells, DEM bioenergetics were lower than NC bioenergetics for Basal (*P* = 0.026), Max (*P* = 0.0012), SRC (*P* = 0.0033), and ATP-linked (*P* = 0.0048) respiration. DEM bioenergetics were also lower than MCI bioenergetics for Max (*P* = 0.083), SRC (*P* = 0.073), and ATP-linked (*P* = 0.092) respiration (Figures 1C–G).

In the high-resolution respirometry assay using permeabilized cells MCI bioenergetics were lower than NC bioenergetics for CI (*P* = 0.008) and Max ETS respiration (*P* = 0.012). DEM bioenergetics were also lower than NC bioenergetics for CI (*P* = 0.011) and Max ETS (*P* = 0.023) respiration (Figures 1I,J).

### Respiration is lower in Neuro-2a cells incubated with DEM plasma compared to cells incubated with NC plasma

In N2a cells treated with plasma MCI bioenergetics were lower than NC bioenergetics for Basal (*P* = 0.098) Max (*P* = 0.002) and SRC (*P* = 0.005) respiration. DEM bioenergetics were also lower than NC bioenergetics for Max (*P* = 0.028) and SRC (*P* = 0.023) respiration (Figures 2C–G). This indicates that plasma contains some circulating factor or factors that have an effect on bioenergetics.

### Naïve cells (Neuro-2a) exposed to plasma exhibit bioenergetic profiles that recapitulate primary cells (PBMCs)

**FIGURE 3:**
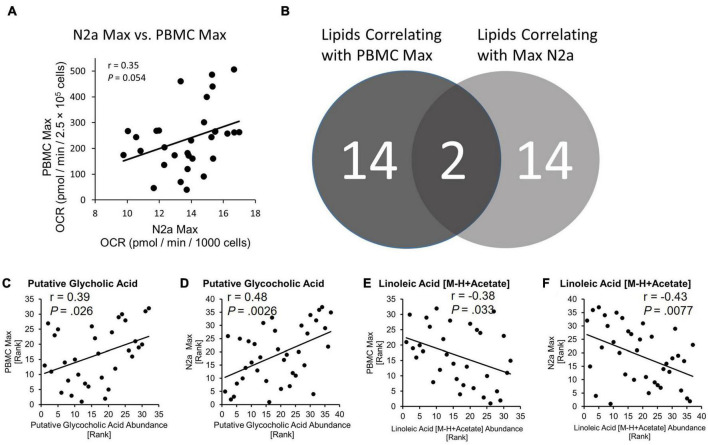
Bioenergetic status of PBMCs is conferred to naïve N2a cells and lipids are related to bioenergetics. **(A)** PBMC Max and Max N2a positively correlate. **(B)** Number of lipids identified as correlating to PBMC Max and N2a Max. Exact *P*-values reported. **(C,D)** Spearman correlation of putative glycocholic acid to PBMC Max and N2a Max. **(E,F)** Spearman correlation of linoleic acid [M-H + Acetate] to PBMC Max and N2a Max.

The relationship between primary PBMC bioenergetics and the bioenergetics of the N2a cells exposed to circulating factors from the fibrinogen-depleted plasma was examined using spearman’s correlation analysis. Maximal bioenergetic capacity, reported by uncoupled Max was preselected as the primary bioenergetic outcome for analysis. PBMC Max and N2a Max values were positively correlated (*r* = 0.35, *P* = 0.054) (Figure 3A).

### Glycocholic acid and linoleic acid identified as candidate mitoactive circulating factors

We used LC-mass spectroscopy to identify and measure the abundance of 197 lipid molecules present in plasma from the discovery cohort (*n* = 37) and the larger validation cohort (*n* = 378). The abundance of lipids from the discovery cohort was then correlated with the values from PBMC Max and N2a Max bioenergetic parameters using Spearman’s correlation. Out of the 197 lipid candidates, 14 had a relationship with PBMC Max and 14 had a relationship with N2a Max (Figure 3B). Two lipids, Putative Glycocholic acid (GCA) and linoleic acid [M-H + Acetate] (LA), correlated with both bioenergetic measures. GCA had a positive relationship with PBMC Max (*r* = 0.39, *P* = 0.026) and N2a Max (*r* = 0.48, *P* = 0.028) (Figures 3C,D) and LA had a negative relationship with PBMC Max (*r* = –0.38, *P* = 0.033) and N2a Max (*r* = –0.43, *P* = 0.0077) (Figures 3E,F).

**FIGURE 4:**
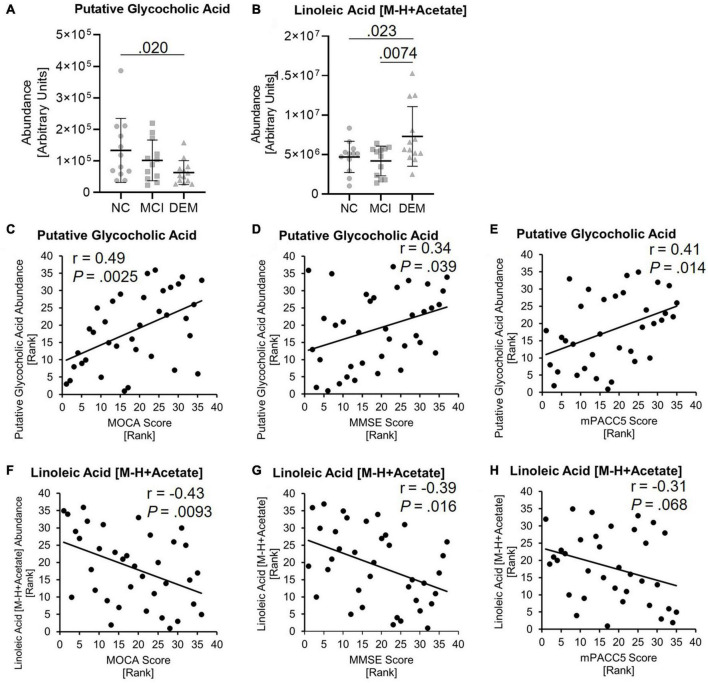
Lipid candidates abundance differs between cognitive groups and correlate with cognitive scores. **(A,B)** Lipid abundance across cognitive groups. **(C–H)** Lipid candidates correlation with MOCA, MMSE, and MPACC5 cognitive scores. Exact *P*-values reported.

GCA abundance was lower by 52.9% in the DEM group as compared to the NC group (*P* = 0.020) in the discovery cohort (Figure 4A) and LA abundance was higher by 35.8% in the DEM group as compared to the NC group (*P* = 0.023) and higher by 42.7% in the DEM group as compared to the MCI group (*P* = 0.0074) (Figure 4B).

GCA had positive relationships with MOCA (*r* = 0.49, *P* = 0.0025), MMSE (*r* = 0.34, *P* = 0.039) and mPACC5 (*r* = 0.41, *P* = 0.014) cognitive scores (Figures 4C–E). In contrast, LA had negative relationships with MOCA (*r* = −0.43, *P* = 0.0093), MMSE (*r* = −0.39, *P* = 0.016) and mPACC5 (*r* = −0.31, *P* = 0.068) cognitive scores (Figures 4F–H).

**FIGURE 5:**
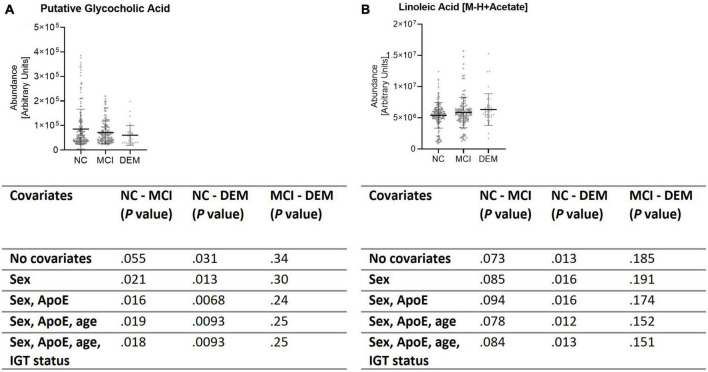
Validation cohort shows same differences in lipid abundance across cognitive groups. **(A,B)** Lipid candidates’ abundance by cognitive group for validation cohort (*n* = 365) and *P*- values for differences between groups when controlled for sex, ApoE status, age, and IGT status.

These findings were validated by evaluating the abundance of GCA and LA in the larger, diverse, cohort (*n* = 365). GCA abundance was lower by 29.7% in the DEM (*P* = 0.012) group and by 16.2% in the MCI group (*P* = 0.025) as compared to the NC group when not controlled for sex, ApoE status (E2/E2, E2/E3, E2/E4, E3/E3, E3/E4, or E4/E4), age, or impaired glucose tolerance (IGT) status (impaired or not impaired). Of note, controlling for ApoE status strengthened the difference between the NC and DEM groups (*P* = 0.0068). The remaining covariates had little to no effect on differences between groups (Figure 5A). LA abundance was greater by 17.8% in the DEM group (*P* = 0.012) and by 8.0% in the MCI group (*P* = 0.11) as compared to the NC group. Controlling for sex, ApoE status, age, or IGT status had little to no effect on differences between groups (Figure 5B).

## Discussion

Mitochondrial alterations occur in the brain and peripheral cells of patients with AD, suggesting systemic bioenergetic decline associated with disease progression. Based on this premise we hypothesized that non-cellular circulating factors, present in plasma, can drive bioenergetic differences based on the cognitive status of older adults. In this study, we demonstrate that both circulating PBMCs, as well as naïve N2a cells exposed to plasma, exhibit lower bioenergetic capacities in DEM compared to NC participants. By combining PBMC and N2a bioenergetic data with lipidomic data, we were able to identify two lipids that have the potential to be “mito-active” and are differentially expressed between NC and DEM cohorts.

The observation of lower PBMC bioenergetics with worse cognitive performance is in agreement with some previous studies examining peripheral bioenergetics in AD. One study examined bioenergetics in PBMCs from 53 patients with mild to moderate probable AD and 30 healthy controls (Maynard et al., 2015). Similar to our results, this study by Maynard et al. (2015) found that Basal respiration in PBMCs was lower in participants with mild to moderate probable AD compared to healthy controls. In contrast, their study did not observe differences in Max, Spare, or ATP linked respiration; however, there were technical differences that should be considered. Their final concentration of FCCP, used to induce Max respiration, was 70% lower than in our study. Suboptimal dosing of FCCP will impact Max respiration and is a major difference between the two studies (Nicholls et al., 2010; Brand and Nicholls, 2011). Differences in blood cell isolation protocols and the composition of the media, especially pyruvate concentration, used during the respirometry assay can also affect results. Another study of 428 healthy controls and 469 MCI participants reported differences in ATP linked respiration (Apaijai et al., 2020). While we did not observe statistically significant differences between NC and MCI groups for ATP linked respiration, our general pattern of data agrees with their findings and we also found no difference between Basal, Leak, Max, or Spare respiration in MCI (Apaijai et al., 2020). Overall, new data on PBMC bioenergetics in the context of dementia provided by this study expands on the current literature by providing additional evidence that peripheral bioenergetics are altered AD dementia.

Seminal studies have shown that blood harbors circulating factors that can mediate the effects of aging (Gan and Südhof, 2019; Pandika, 2019). Our lab has previously demonstrated that parabiosis of young and old mice leads to significant bioenergetic decline for the young parabionts (Gonzalez-Armenta et al., 2021). A study from another group demonstrated that young plasma reduced AD pathology and improved cognition in mice, providing evidence that factors in the plasma affect brain pathology (Zhao et al., 2020). The study presented here is the first to examine and compare the effects of plasma from NC, MCI, and DEM individuals on naïve N2a bioenergetics. Our data revealed that circulating factors differ between cognitive groups and that plasma from participants with DEM can have inhibitory effect on mitochondrial function.

We found that mitochondrial bioenergetics of PBMCs and N2a cells exposed to plasma from the PBMC donor are positively correlated. This finding suggests that factors present in plasma can influence mitochondrial function in the same manner, across multiple cell types, both *in vivo* and *in vitro*.

Based on these results, we aimed to identify circulating lipid candidates with the potential to be mito-active. Among the various classes of circulating factors, lipids are of particular interest. Circulating lipids have been used to identify healthy controls from AD patients (Proitsi et al., 2015) and are linked to amyloid-β accumulation AD risk genes (Liu et al., 2021). We performed LC-MS on plasma samples to quantify the abundance of known 197 lipids. In order to identify candidate molecules that can directly affect mitochondrial function according to the cognitive status, we utilized two primary selection criteria. First, the abundance of the molecule had to be correlated with the bioenergetic capacity of PBMCs from the plasma donor. Second, the abundance of the molecule had to be correlated with the bioenergetic capacity of N2a cells exposed to the corresponding plasma sample. Using these two criteria, we are better able identify candidate mito-active molecules with direct bioenergetic effects. One of the candidate molecules meeting both selection criteria, GCA, was decreased in the DEM group and positively correlated with three cognitive performance measures. GCA is a conjugated bile acid derived from glycine and cholic acid. Although there are no direct studies examining GCA blood-brain barrier permeability, bile acids are generally accepted as blood-brain barrier permeable. Some bile acids, such as tauroursodeoxycholic acid and ursodeoxycholic acid, are not only known to cross the blood-brain barrier but have been studied as therapeutic agents to alleviate neurodegenerative disorders (Rodrigues et al., 2003; Parry et al., 2010; Ackerman and Gerhard, 2016). Bile acids have also been shown to stabilize mitochondrial membranes, decrease free radical formation, inhibit mitochondrial permeability transition, impair apoptotic pathways, increase activity of mitochondrial complexes I-IV, and rescue mitochondrial and cell functions in disease models (Rodrigues et al., 2000; Keene et al., 2001; Mortiboys et al., 2013). As bile acids are already being investigated as neurotherapeutics and our data shows that GCA is decreased in individuals with dementia due to probable AD, this makes GCA a prime target for further investigation. If GCA has stimulatory bioenergetic effects in further experiments, GCA could have potential as a mitochondrial therapeutic (Ackerman and Gerhard, 2016).

Another molecule meeting both selection criteria is LA. Our analyses indicate that LA is increased in the DEM group compared to the NC and MCI groups and is negatively correlated with three cognitive scores. LA is an essential polyunsaturated omega-6 fatty acid found in a variety of foods but is especially prevalent in soybean oil, nuts, seed, meat, and eggs. Although linoleic acid is found in many nutrient rich foods and is associated with positive health outcomes, it is also highly abundant in processed foods that use soybean oil, therefore and elevated consumption may not always be associated with a health. As our cohort is expected to primarily consume a western diet, this is of particular importance to consider. LA is blood-brain-barrier penetrable, however, most of the LA that enters the brain is not converted to AA (DeMar et al., 2006; Chen et al., 2008). Roughly 59% of LA transported into the brain is broken down by fatty acid β-oxidation and results in metabolites that are likely products of the TCA cycle (DeMar et al., 2006). Fatty acid β-oxidation is not the preferred pathway to generate energy in the brain due to elevated ROS production, against which neurons have poor defenses (Chen et al., 2014). Excessive ROS production is detrimental to mitochondrial function and could be a way that LA plays a role in decreased bioenergetic function in AD. Other studies have also found that lowering LA consumption attenuates migraine symptoms, increases anti-inflammatory metabolites, and reduces pro-inflammatory eicosanoids derived from AA (Lin et al., 2015; Ramsden et al., 2015; Taha et al., 2018). As LA crosses the blood-brain barrier, has a potential mechanism of impairment toward CNS cells, and decreased consumption is thought to reduce inflammation and relieve CNS symptoms, this indicates that LA is also an ideal target for further investigation into potential bioenergetic effects and as a potential mediator of dementia-related bioenergetic decline.

A key feature of this study is the validation of GCA and LA in a large 378 participant cohort. We utilized this larger cohort to demonstrate that our screening approach can identify individual factors on a small scale and extend the findings to a larger diverse population. As anticipated, our data indicate that GCA is lower and LA is higher in the DEM compared to NC participants in the larger cohort. It is notable that the *p*-values are enhanced in the larger cohort except for between LA levels between the MCI and DEM groups. It should be noted that the smaller discovery cohort was well balanced in age, BMI, and sample number across cognitive groups. There were well defined differences in cognitive scores between the NC and DEM groups as well as the MCI and DEM groups. The NC and MCI groups were less defined, however, the cognitive status was determined by other factors as well, such as MRI and clinical evaluation. Moreover, the cohort was designed to include only normoglycemic individuals. The validation cohort was well balanced in BMI and had distinct differences in cognitive scores between cognitive groups. However, there was a difference in average age between the cognitive groups in this larger cohort (Table 2). Moreover, the percentage of the cohort representing the DEM group compared to the NC and MCI groups was considerably lower, leading to an imbalance in the group distribution (Table 2). The validation cohort also included all individuals in the study regardless of IGT status. When examining difference between groups in the validation cohort we use sex, ApoE status, age, and IGT status as covariates. Of note, controlling for sex and sex with ApoE status strengthened the difference between the NC and DEM groups for the GCA comparisons. Controlling for sex moderately strengthened the differences while adding ApoE status as a covariate had a robust effect. ApoE is a lipoprotein that transports fats in the blood and individuals with the ApoE4 allele are at greater risk for developing AD. These findings indicated the GCA levels and ApoE status are related. Despite the limitations and variability of the larger validation cohort, differences in the abundances of candidate lipids were still significant and reflective of the discovery cohort. Another limitation of this study was grouping individuals based on cognitive status rather than AD biomarkers. The MCI group in particular contains individuals who will progress to AD and individuals who are impaired for other reasons and may or may not progress to dementia.

Another limitation was incomplete data HbA1c data for both the discovery and validation cohorts. The discovery cohort was intentionally selected to include only normoglycemic individuals and the validation cohort included both normoglycemic and individuals with impaired glucose tolerance. There was a relatively even balance of normoglycemic compared to impaired glucose tolerance individuals for all three groups with 53, 54, and 55% of the NC, MCI, and DEM groups, respectively, that were normoglycemic. Additionally, HbA1c is a good indicator of average glucose levels over the past 2–3 months but does not provide short term changes in blood glucose. Another point to consider is the nutritional status of the participants as diet is likely to affect plasma lipid composition. As 82% of our study population is Caucasian, the diet of these participants is expected to be primarily a western diet, which is known to be high in saturated fats, although we do not have data on specific food items (Cordain et al., 2005). The western diet is a risk factor for Alzheimer’s disease and is known to disrupt mitochondrial bioenergetics (Grant, 2016; Amick et al., 2021). Therefore, this population is likely to be susceptible to the influence of saturated fats on bioenergetics.

Future work should explore the direct effects of GCA and LA on neuronal bioenergetics by exposing neurons to the lipids. Although bioenergetics effects are likely a concerted effect of circulating factors working together, identifying the effects of singular components provides insight into how the cumulative effect occurs. Additionally, exposing neurons to individual lipids should be tried on additional cell lines. Immortal cell lines are useful for screening assays, but more detailed work should use human derived cells. Future should also include utilizing publicly available lipidomic datasets to validate our findings in other large clinical studies. Additional experiments that could be utilized to further understand the effect of lipids on bioenergetics would be assays to measure aspects of mitochondrial physiology that may affect bioenergetics such as changes in number, size, shape, mitochondrial quality control (e.g., fusion and fission), and mitochondrial content (e.g., biogenesis and degradation).

In conclusion, these results are the first to demonstrate that total plasma factors have an overall inhibitory effect on bioenergetics in individuals with cognitive impairment. Furthermore, our screening approach was able to identify two candidate lipids that have the potential to be mito-active compounds in the plasma samples from our participants. Validation of our two lipid candidates in a larger cohort demonstrates the power of this approach. Importantly, our approach can be adopted for use in other biorepositories and for the identification of other types of circulating factors present in blood, thus creating new opportunities for future studies of existing samples and data.

## Data availability statement

The raw data supporting the conclusions of this article will be made available by the authors, without undue reservation.

## Ethics statement

The studies involving human participants were reviewed and approved by the Wake Forest School of Medicine Institutional Review Board. The patients/participants provided their written informed consent to participate in this study.

## Author contributions

KA, MJ, SC, and AM contributed to the conception and design of the study. KA, GM, ZG, and AD performed biological assays. KA performed the statistical analysis and wrote the first draft of the manuscript. KA and AM revised the manuscript. All authors read and approved the submitted version.

## Abbreviations

AD: Alzheimer’s disease
ARC: Alzheimer’s disease Clinical Core
DEM: Dementia due to probable Alzheimer’s disease
DSST: Digit Symbol Substitution Task
FCSRT: Free and Cued Selective Reminding Test
GCA: Putative glycocholic acid
IGT: Impaired glucose tolerance
LA: Linoleic acid [M-H + Acetate]
MCI: Mild cognitive impairment
N2a: Neuro 2a neuronal cell line
MMSE: Mini-Mental State Exam
mPACC5: Modified preclinical Alzheimer’s cognitive composite
PACC5: Preclinical Alzheimer’s cognitive composite
UDS: National Alzheimer’s Disease Centers’ Uniform Data Set version 3
WF ADRC: Wake Forest Alzheimer’s Disease Research Center.
