# Supplementary material for: Plasma glycocholic acid and linoleic acid identified as potential mediators of mitochondrial bioenergetics in Alzheimer’s dementia
Source: Front Aging Neurosci. 2022 Sep 23;14:954090. doi: 10.3389/fnagi.2022.954090 (PMC9540364; doi:10.3389/fnagi.2022.954090)
Supplement: Supplementary file 1 [file Table_1.DOCX]

| Supplementary Table 1: Pairwise Comparisons of the Discovery Cohort Demographics | | | |
| --- | --- | --- | --- |
|  | **Mean Difference (*P* value)** | | |
|  | **NC vs. MCI** | **NC vs. DEM** | **MCI vs. DEM** |
| Age, y | 0.42 (.980) | -0.62 (.954) | -1.04 (.878) |
| BMI | -1.19 (.735) | -1.26 (.699) | -0.07 (.999) |
| MMSE, score | 0.67 (.863) | 6.82 (<.0001) | 6.15 (<.0001) |
| MOCA, score | 2.83 (.061) | 8.17 (<.0001) | 5.33 (.0003) |
| mPACC5, score | 0.93 (.421) | 4.81 (<.0001) | 3.88 (<.0001) |
| HbA1c | -0.03 (.990) | -0.12 (.849) | -0.09 (.857) |

HbA1c, Hemoglobin A1c
For age, BMI, MMSE, MOCA, and mPACC5 parameters: NC, n = 12; MCI, n = 12; DEM, n = 13
For HbA1c parameter: NC, n = 4; MCI, n = 9; DEM, n = 11

| Supplementary Table 2: Pairwise Comparisons of the Validation Cohort Demographics | | | |
| --- | --- | --- | --- |
|  | **Mean Difference (*P* value)** | | |
|  | **NC vs. MCI** | **NC vs. DEM** | **MCI vs. DEM** |
| Age, y | -2.53 (.011) | -5.87 (<.0001) | -3.34 (.050) |
| BMI | -0.36 (.828) | 1.23 (.423) | 1.60 (.259) |
| MMSE, score | 1.53 (<.0001) | 5.88 (<.0001) | 4.35 (<.0001) |
| MOCA, score | 4.61 (<.0001) | 7.45 (<.0001) | 2.84 (.004) |
| mPACC5, score | 1.25 (<.0001) | 4.52 (<.0001) | 3.27 (<.0001) |
| HbA1c | -0.16 (.128) | -0.0029 (.9997) | 0.16 (.384) |

HbA1c, Hemoglobin A1c
For age, BMI, MMSE, MOCA, and mPACC5 parameters: NC, n = 188; MCI, n = 138; DEM, n = 39
For HbA1c parameter: NC, n = 103, MCI = 91, and DEM = 31.
